# 4-Nitro-*N*^2^-(pyridin-4-ylmethylidene)benzene-1,2-diamine

**DOI:** 10.1107/S1600536813015481

**Published:** 2013-06-12

**Authors:** Guang-Lin Liu, Jun Sun, Jing-Chao Zhang, Jie Mei, Cheng Guo

**Affiliations:** a College of Science, Nanjing University of Technology, Xinmofan Road No.5 Nanjing, Nanjing 210009, People’s Republic of China

## Abstract

In the title compound, C_12_H_10_N_4_O_2_, the dihedral angle between the aromatic rings is 43.18 (16)°. The nitro group is rotated from its attached ring by 7.8 (2)° and a short intra­molecular N—H⋯N contact occurs. In the crystal, the mol­ecules are linked by N—H⋯N and C—H⋯O hydrogen bonds, generating a three-dimensional network.

## Related literature

For the synthesis, see: Luo *et al.* (2009 ▶).
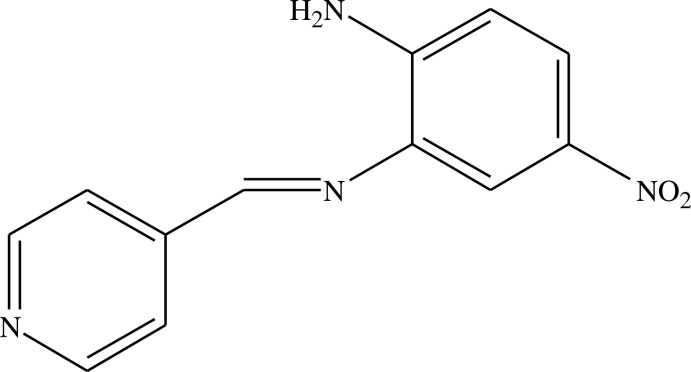


## Experimental

### 

#### Crystal data

C_12_H_10_N_4_O_2_
*M* *_r_* = 242.24
Monoclinic, 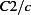

*a* = 21.324 (4) Å
*b* = 9.1480 (18) Å
*c* = 12.950 (3) Å
β = 116.36 (3)°
*V* = 2263.5 (8) Å^3^
*Z* = 8
Mo *K*α radiation
μ = 0.10 mm^−1^
*T* = 293 K
0.30 × 0.20 × 0.10 mm

#### Data collection

Enraf–Nonius CAD-4 diffractometer
Absorption correction: ψ scan (North *et al.*, 1968 ▶) *T*_min_ = 0.970, *T*_max_ = 0.990
2128 measured reflections
2070 independent reflections
1247 reflections with *I* > 2σ(*I*)
*R*_int_ = 0.038
3 standard reflections every 200 reflections intensity decay: 1%

#### Refinement

*R*[*F* ^2^ > 2σ(*F* ^2^)] = 0.058
*wR*(*F* ^2^) = 0.175
*S* = 1.01
2070 reflections
164 parameters
H-atom parameters constrained
Δρ_max_ = 0.19 e Å^−3^
Δρ_min_ = −0.18 e Å^−3^

### 

Data collection: *CAD-4 EXPRESS* (Enraf–Nonius, 1994) ▶; cell refinement: *CAD-4 EXPRESS*; data reduction: *XCAD4* (Harms & Wocadlo, 1995 ▶); program(s) used to solve structure: *SHELXS97* (Sheldrick, 2008 ▶); program(s) used to refine structure: *SHELXL97* (Sheldrick, 2008 ▶); molecular graphics: *SHELXTL* (Sheldrick, 2008 ▶); software used to prepare material for publication: *PLATON* (Spek, 2009) ▶.

## Supplementary Material

Crystal structure: contains datablock(s) D, I. DOI: 10.1107/S1600536813015481/hb7085sup1.cif

Structure factors: contains datablock(s) I. DOI: 10.1107/S1600536813015481/hb7085Isup2.hkl

Additional supplementary materials:  crystallographic information; 3D view; checkCIF report

## Figures and Tables

**Table 1:** Hydrogen-bond geometry (Å, °)

*D*—H⋯*A*	*D*—H	H⋯*A*	*D*⋯*A*	*D*—H⋯*A*
N2—H2*A*⋯N4^i^	0.86	2.42	3.091 (3)	135
N2—H2*B*⋯N3	0.86	2.42	2.751 (3)	103
C10—H10*A*⋯O1^ii^	0.93	2.49	3.156 (5)	128

Symmetry codes: (i) 
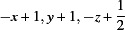
; (ii) 
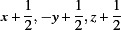
.

**Fig. 1.**
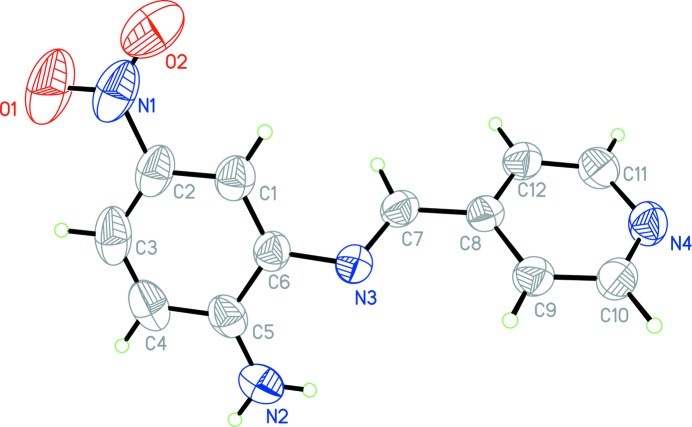
The molecular structure of the title molecule, with displacement ellipsoids drawn at the 50% probability level.

**Fig. 2.**
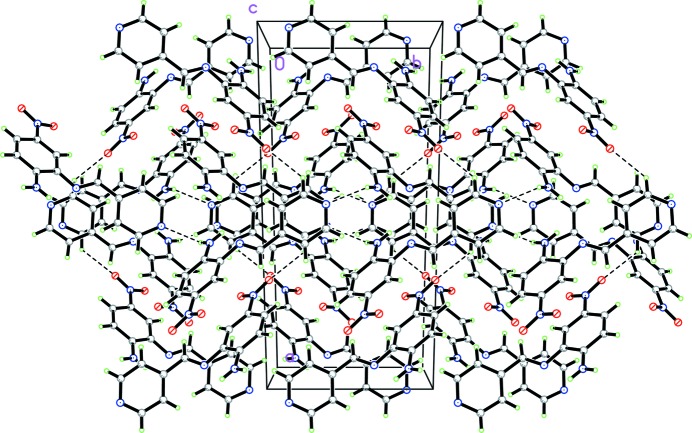
A packing diagram of the title molecule.

## supplementary crystallographic information

### Experimental

The title compound, (I), which may have applications as a metal fluoresence probe, was prepared by the literature method (Luo *et al.*, 2009). Yellow blocks were obtained by dissolving (I) (0.18 g, 1.0mmol) in ethanol (25 ml) and evaporating the solvent slowly at room temperature.

### Refinement

H atoms were positioned geometrically and refined as riding groups, with N—H = 0.86 and C—H = 0.93 Å for aromatic H, and constrained to ride on their parent atoms, with *U*_iso_(H) = *xU*_eq_(C), where *x* = 1.2 for aromatic H, and *x* = 1.5 for other H.

### Crystal data

**Table:**

C_12_H_10_N_4_O_2_	*D*_x_ = 1.422 Mg m^−^^3^
*M**_r_* = 242.24	Melting point: 449.65 K
Monoclinic, *C*2/*c*	Mo *K*α radiation, λ = 0.71073 Å
*a* = 21.324 (4) Å	Cell parameters from 25 reflections
*b* = 9.1480 (18) Å	θ = 10–13°
*c* = 12.950 (3) Å	µ = 0.10 mm^−^^1^
β = 116.36 (3)°	*T* = 293 K
*V* = 2263.5 (8) Å^3^	Block, yellow
*Z* = 8	0.30 × 0.20 × 0.10 mm
*F*(000) = 1008	

### Data collection

**Table:**

Enraf–Nonius CAD-4 diffractometer	1247 reflections with *I* > 2σ(*I*)
Radiation source: fine-focus sealed tube	*R*_int_ = 0.038
Graphite monochromator	θ_max_ = 25.4°, θ_min_ = 2.1°
ω/2θ scans	*h* = 0→25
Absorption correction: ψ scan (North *et al.*, 1968)	*k* = 0→11
*T*_min_ = 0.970, *T*_max_ = 0.990	*l* = −15→14
2128 measured reflections	3 standard reflections every 200 reflections
2070 independent reflections	intensity decay: 1%

### Refinement

**Table:**

Refinement on *F*^2^	Secondary atom site location: difference Fourier map
Least-squares matrix: full	Hydrogen site location: inferred from neighbouring sites
*R*[*F*^2^ > 2σ(*F*^2^)] = 0.058	H-atom parameters constrained
*wR*(*F*^2^) = 0.175	*w* = 1/[σ^2^(*F*_o_^2^) + (0.090*P*)^2^] where *P* = (*F*_o_^2^ + 2*F*_c_^2^)/3
*S* = 1.01	(Δ/σ)_max_ < 0.001
2070 reflections	Δρ_max_ = 0.19 e Å^−^^3^
164 parameters	Δρ_min_ = −0.18 e Å^−^^3^
0 restraints	Extinction correction: *SHELXL*, Fc^*^=kFc[1+0.001xFc^2^λ^3^/sin(2θ)]^-1/4^
Primary atom site location: structure-invariant direct methods	Extinction coefficient: 0.0052 (12)

### Special details

**Table:**

Geometry. All esds (except the esd in the dihedral angle between two l.s. planes) are estimated using the full covariance matrix. The cell esds are taken into account individually in the estimation of esds in distances, angles and torsion angles; correlations between esds in cell parameters are only used when they are defined by crystal symmetry. An approximate (isotropic) treatment of cell esds is used for estimating esds involving l.s. planes.
Refinement. Refinement of F^2^ against ALL reflections. The weighted R-factor wR and goodness of fit S are based on F^2^, conventional R-factors R are based on F, with F set to zero for negative F^2^. The threshold expression of F^2^ > 2sigma(F^2^) is used only for calculating R-factors(gt) etc. and is not relevant to the choice of reflections for refinement. R-factors based on F^2^ are statistically about twice as large as those based on F, and R- factors based on ALL data will be even larger.

### Fractional atomic coordinates and isotropic or equivalent isotropic displacement parameters (Å^2^)

**Table:**

	*x*	*y*	*z*	*U*_iso_*/*U*_eq_	
C1	0.32477 (14)	0.2795 (3)	−0.0542 (3)	0.0521 (8)	
H1A	0.3229	0.2145	−0.1108	0.062*	
N1	0.23039 (15)	0.4169 (4)	−0.2041 (3)	0.0792 (10)	
O1	0.18599 (16)	0.5126 (4)	−0.2272 (3)	0.1194 (12)	
N2	0.42131 (15)	0.3378 (3)	0.2557 (2)	0.0666 (8)	
H2A	0.4219	0.3980	0.3072	0.080*	
H2B	0.4499	0.2652	0.2754	0.080*	
C2	0.27963 (16)	0.3970 (4)	−0.0840 (3)	0.0596 (9)	
O2	0.23537 (15)	0.3379 (4)	−0.2766 (3)	0.0991 (10)	
N3	0.42154 (12)	0.1420 (2)	0.09465 (19)	0.0461 (6)	
C3	0.28139 (17)	0.4952 (4)	−0.0015 (4)	0.0714 (11)	
H3B	0.2506	0.5740	−0.0223	0.086*	
C4	0.32854 (17)	0.4753 (3)	0.1102 (4)	0.0664 (10)	
H4A	0.3297	0.5414	0.1656	0.080*	
N4	0.53086 (14)	−0.3605 (2)	0.1282 (2)	0.0546 (7)	
C5	0.37562 (15)	0.3574 (3)	0.1443 (3)	0.0526 (8)	
C6	0.37257 (14)	0.2579 (3)	0.0586 (2)	0.0455 (7)	
C7	0.40423 (15)	0.0216 (3)	0.0405 (2)	0.0472 (7)	
H7A	0.3597	0.0141	−0.0205	0.057*	
C8	0.45009 (14)	−0.1054 (3)	0.0682 (2)	0.0440 (7)	
C9	0.52046 (15)	−0.1025 (3)	0.1456 (2)	0.0475 (8)	
H9A	0.5420	−0.0148	0.1789	0.057*	
C10	0.55786 (15)	−0.2301 (3)	0.1725 (2)	0.0501 (7)	
H10A	0.6050	−0.2258	0.2247	0.060*	
C11	0.46362 (17)	−0.3624 (3)	0.0523 (3)	0.0578 (9)	
H11A	0.4435	−0.4513	0.0194	0.069*	
C12	0.42246 (16)	−0.2395 (3)	0.0201 (3)	0.0541 (8)	
H12A	0.3759	−0.2464	−0.0341	0.065*	

### Atomic displacement parameters (Å^2^)

**Table:**

	*U*^11^	*U*^22^	*U*^33^	*U*^12^	*U*^13^	*U*^23^
C1	0.0439 (16)	0.0497 (17)	0.0602 (19)	0.0008 (14)	0.0209 (15)	0.0038 (15)
N1	0.0479 (17)	0.081 (2)	0.093 (3)	0.0097 (17)	0.0174 (18)	0.035 (2)
O1	0.0750 (18)	0.108 (2)	0.143 (3)	0.0420 (18)	0.0195 (18)	0.053 (2)
N2	0.0734 (19)	0.0603 (17)	0.0600 (18)	−0.0034 (15)	0.0241 (15)	−0.0172 (14)
C2	0.0397 (16)	0.056 (2)	0.075 (2)	0.0068 (15)	0.0181 (16)	0.0182 (17)
O2	0.0711 (19)	0.129 (3)	0.072 (2)	0.0121 (17)	0.0095 (15)	0.0210 (18)
N3	0.0456 (14)	0.0417 (14)	0.0456 (14)	0.0029 (11)	0.0154 (11)	0.0011 (11)
C3	0.0437 (18)	0.047 (2)	0.118 (3)	0.0074 (15)	0.031 (2)	0.014 (2)
C4	0.057 (2)	0.0462 (19)	0.102 (3)	−0.0031 (16)	0.041 (2)	−0.0124 (19)
N4	0.0548 (16)	0.0442 (15)	0.0627 (17)	0.0053 (12)	0.0242 (14)	0.0004 (12)
C5	0.0489 (17)	0.0417 (17)	0.070 (2)	−0.0083 (14)	0.0285 (17)	−0.0072 (15)
C6	0.0416 (15)	0.0404 (15)	0.0510 (17)	−0.0003 (13)	0.0174 (14)	0.0020 (13)
C7	0.0415 (15)	0.0455 (17)	0.0451 (16)	−0.0009 (13)	0.0106 (13)	0.0002 (13)
C8	0.0461 (16)	0.0438 (17)	0.0389 (15)	0.0014 (13)	0.0160 (13)	0.0006 (12)
C9	0.0471 (17)	0.0437 (17)	0.0465 (17)	−0.0015 (13)	0.0159 (14)	−0.0017 (13)
C10	0.0442 (16)	0.0512 (18)	0.0503 (17)	0.0023 (14)	0.0170 (13)	0.0015 (14)
C11	0.064 (2)	0.0414 (18)	0.063 (2)	−0.0051 (16)	0.0235 (17)	−0.0064 (15)
C12	0.0486 (17)	0.0504 (19)	0.0527 (17)	−0.0013 (15)	0.0129 (14)	−0.0052 (15)

### Geometric parameters (Å, º)

**Table:**

C1—C6	1.375 (4)	C4—H4A	0.9300
C1—C2	1.379 (4)	N4—C11	1.331 (4)
C1—H1A	0.9300	N4—C10	1.338 (3)
N1—O1	1.225 (4)	C5—C6	1.414 (4)
N1—O2	1.226 (4)	C7—C8	1.457 (4)
N1—C2	1.450 (5)	C7—H7A	0.9300
N2—C5	1.347 (4)	C8—C12	1.383 (4)
N2—H2A	0.8600	C8—C9	1.386 (4)
N2—H2B	0.8600	C9—C10	1.368 (4)
C2—C3	1.384 (5)	C9—H9A	0.9300
N3—C7	1.269 (3)	C10—H10A	0.9300
N3—C6	1.414 (3)	C11—C12	1.372 (4)
C3—C4	1.359 (5)	C11—H11A	0.9300
C3—H3B	0.9300	C12—H12A	0.9300
C4—C5	1.405 (4)		
			
C6—C1—C2	120.4 (3)	C4—C5—C6	118.1 (3)
C6—C1—H1A	119.8	C1—C6—C5	119.7 (3)
C2—C1—H1A	119.8	C1—C6—N3	123.3 (3)
O1—N1—O2	123.7 (4)	C5—C6—N3	117.0 (2)
O1—N1—C2	117.7 (4)	N3—C7—C8	123.8 (3)
O2—N1—C2	118.6 (3)	N3—C7—H7A	118.1
C5—N2—H2A	120.0	C8—C7—H7A	118.1
C5—N2—H2B	120.0	C12—C8—C9	116.8 (3)
H2A—N2—H2B	120.0	C12—C8—C7	119.4 (2)
C1—C2—C3	120.9 (3)	C9—C8—C7	123.7 (3)
C1—C2—N1	118.7 (4)	C10—C9—C8	119.3 (3)
C3—C2—N1	120.3 (3)	C10—C9—H9A	120.4
C7—N3—C6	118.5 (2)	C8—C9—H9A	120.4
C4—C3—C2	119.3 (3)	N4—C10—C9	124.1 (3)
C4—C3—H3B	120.4	N4—C10—H10A	118.0
C2—C3—H3B	120.4	C9—C10—H10A	118.0
C3—C4—C5	121.7 (3)	N4—C11—C12	123.2 (3)
C3—C4—H4A	119.2	N4—C11—H11A	118.4
C5—C4—H4A	119.2	C12—C11—H11A	118.4
C11—N4—C10	116.5 (2)	C11—C12—C8	120.2 (3)
N2—C5—C4	120.9 (3)	C11—C12—H12A	119.9
N2—C5—C6	121.0 (3)	C8—C12—H12A	119.9
			
C6—C1—C2—C3	0.0 (5)	N2—C5—C6—N3	−2.8 (4)
C6—C1—C2—N1	179.5 (3)	C4—C5—C6—N3	178.6 (2)
O1—N1—C2—C1	172.5 (3)	C7—N3—C6—C1	−32.3 (4)
O2—N1—C2—C1	−7.6 (5)	C7—N3—C6—C5	149.7 (3)
O1—N1—C2—C3	−8.0 (5)	C6—N3—C7—C8	179.3 (2)
O2—N1—C2—C3	171.9 (3)	N3—C7—C8—C12	167.2 (3)
C1—C2—C3—C4	0.3 (5)	N3—C7—C8—C9	−9.4 (4)
N1—C2—C3—C4	−179.2 (3)	C12—C8—C9—C10	−1.7 (4)
C2—C3—C4—C5	0.0 (5)	C7—C8—C9—C10	175.0 (3)
C3—C4—C5—N2	−178.9 (3)	C11—N4—C10—C9	1.1 (4)
C3—C4—C5—C6	−0.4 (4)	C8—C9—C10—N4	0.2 (4)
C2—C1—C6—C5	−0.4 (4)	C10—N4—C11—C12	−0.7 (5)
C2—C1—C6—N3	−178.3 (3)	N4—C11—C12—C8	−0.9 (5)
N2—C5—C6—C1	179.1 (3)	C9—C8—C12—C11	2.0 (4)
C4—C5—C6—C1	0.6 (4)	C7—C8—C12—C11	−174.8 (3)

### Hydrogen-bond geometry (Å, º)

**Table:**

*D*—H···*A*	*D*—H	H···*A*	*D*···*A*	*D*—H···*A*
N2—H2*A*···N4^i^	0.86	2.42	3.091 (3)	135
N2—H2*B*···N3	0.86	2.42	2.751 (3)	103
C10—H10*A*···O1^ii^	0.93	2.49	3.156 (5)	128

Symmetry codes: (i) −*x*+1, *y*+1, −*z*+1/2; (ii) *x*+1/2, −*y*+1/2, *z*+1/2.
